# Roles of TLR/MyD88/MAPK/NF-κB Signaling Pathways in the Regulation of Phagocytosis and Proinflammatory Cytokine Expression in Response to *E*. *faecalis* Infection

**DOI:** 10.1371/journal.pone.0136947

**Published:** 2015-08-28

**Authors:** Jun Zou, Nathan Shankar

**Affiliations:** Department of Pharmaceutical Sciences, University of Oklahoma Health Sciences Center, Oklahoma City, Oklahoma, United States of America; Institut National de la Santé et de la Recherche Médicale (INSERM), FRANCE

## Abstract

*Enterococcus faecalis* is a commensal bacterium residing in the gastrointestinal tract of mammals, but in certain situations it is also an opportunistic pathogen which can cause serious disease. Macrophages have been shown to play a critical role in controlling infections by commensal enterococci and also have an important role in mediating chromosomal instability and promoting colon cancer during high-level enterococcal colonization in genetically susceptible mice. However, the molecular mechanisms involved in the interaction of macrophages with enterococci during infection are not fully understood. In this study, using BMDM and RAW264.7 macrophages we show that enterococcal infection activates ERK, JNK and p38 MAPK as well as NF-κB, and drives polarization of macrophages towards the M1 phenotype. Inhibition of NF-κB activation significantly reduced the expression of TNF-α and IL-1β, as did the inhibition of ERK, JNK and p38 MAPK, although to differing extent. Enterococci-induced activation of these pathways and subsequent cytokine expression was contact dependent, modest compared to activation by *E*. *coli* and, required the adaptor protein MyD88. Phagocytosis of enterococci by macrophages was enhanced by preopsonization with *E*. *faecalis* antiserum and involved the ERK and JNK signaling pathways, with the adaptor protein MyD88 as an important mediator. This study of the interaction of macrophages with enterococci could provide a foundation for studying the pathogenesis of infection by this opportunistic pathogen and to developing new therapeutic approaches to combat enterococcal infection.

## Introduction

Enterococci are commensal organisms colonizing the gastrointestinal tract of all human and animals [1]. Despite being a commensal organism, *E*. *faecalis* is endowed with traits that make it an opportunistic pathogen, especially in the immunocompromised host. Manifestations of enterococcal disease include urinary tract infections, hepatobiliary sepsis, endocarditis, surgical wound infections, bacteremia and neonatal sepsis [2]. In critically ill hospitalized patients, enterococci frequently produce severe infection which often leads to sepsis and death [3,4]. Furthermore, treatment options for these infections can often be an insurmountable challenge in the clinical setting on account of the high levels of intrinsic antibiotic resistance encountered in *E*. *faecalis* and *E*. *faecium*, the two most common enterococcal species associated with infections. Despite the clinical importance of enterococci, little is known about innate defense mechanisms that protect the normal host against invasive enterococcal infections.

Colonization of the gastrointestinal tract and infection of systemic sites such as the spleen and liver involves multifaceted interactions between bacteria and macrophages [5]. Macrophages are efficient in killing of bacteria through phagocytosis, induction of inflammatory cytokine production and initiation of the adaptive immune response. One of the first studies addressing the contribution of specific innate immune cells to the early control of enterococcal infection was conducted by depletion of macrophages using clodronate-encapsulated liposomes. This study showed that peritoneal macrophages are important in the early containment of *E*. *faecium* peritonitis [6]. By using interleukin 10 (IL-10) deficient mice as a model, recent studies have shown that *Enterococcus faecalis* can causes intestinal inflammation and colon cancer during long-term colonization. During this process macrophages act as key effectors with bystander effects in the progression of cancer formation [7,8]. However, the immunobiology of enterococcus-induced inflammatory responses in macrophages remain to be fully understood.

Upon microorganism invasion, resident macrophages or those recruited to the infection site, phagocytize the pathogen relying on specific receptors such as mannose receptor, or Fc and complement receptors [9]. Engagement of these receptors induces specific signaling pathways and cytoskeletal changes that lead to internalization of the pathogen. Meanwhile, the interaction of Toll-like receptors with pathogen associated molecular patterns (PAMP) activates a variety of signal transduction pathways, leading to the production of inflammatory cytokines and initiation of downstream inflammatory cascades [10]. Among the critical pathways mediating the inflammatory response in eukaryotes is the MAPK system, comprising the extracellular signal-regulated kinases (ERKs) (ERK1/2 or p42/p44), the c-Jun N-terminal kinases (JNKs) (JNK/SAPK) and the p38 MAPK [11]. The activation of MAPKs can phosphorylate other substrates and transcription factors. One of widely-studied transcriptional factors which lies downstream of the MAPK signaling pathway is NF-κB (nuclear transcription factor kappa B), which can activate the transcription of proinflammatory genes or cytoskeletal proteins [12]. Proinflammatory cytokines in turn mediate a number of physiologic changes characteristic of the inflammatory response including recruitment and activation of monocytes and other phagocytes, effecting a variety of changes in organs and tissues and activation of the adaptive immune system [13]. In addition to releasing cytokines to alter host physiology in response to infection, macrophages are responsible for the earliest phases of fending off infection through the killing and processing of microbes. Intracellular killing of bacteria is accomplished through the generation of reactive oxygen and nitrogen species within the phagolysosome by enzymes such as myeloperoxidase MPO and inducible NO synthase (iNOS), which in turn lyse bacterial cells [14].

The effects of NF-κB and MAPKs on phagocytosis and inflammation during enterococcal infection are only now beginning to be investigated. In this study, we infected macrophages from wild type, TLR2^-/-^ and MyD88^-/-^ mice with a clinical *E*. *faecalis* isolate, E99, and examined key aspects of enterococcal-host interactions including phagocytosis, macrophage polarization, proinflammatory cytokine expression and the host signaling pathways involved.

## Materials and Methods

Animal procedures limited to isolation of bone marrow derived macrophages were carried out in accordance with the guidelines set forth by the Public Health Service Policy on Humane Care and Use of Laboratory Animals. The University of Oklahoma Health Sciences Center Institutional Animal Care and Use Committee approved the protocol (15-005-HI) used in this work. Isoflurane was used for anesthesia/euthanasia and efforts were made to minimize suffering.

### Bacterial strains and growth conditions

*Enterococcus faecalis* E99 strain is a clinical isolate from the urine of a patient [15]. *Escherichia coli* TOP10 cells were obtained from Invitrogen. The *E*. *faecalis* and *E*. *coli* stains were grown in THB containing 1% glucose and Luria-Bertani (LB) broth supplemented with the appropriate antibiotics, respectively, for 16 h. To prepare killed *E*. *faecalis*, 4% paraformaldehyde was used. The bacteria were pelleted by centrifugation and washed with phosphate buffered saline (PBS) before use.

### Cell culture and infection

Bone marrow-derived macrophages (BMDM) were isolated from WT, TLR2^-/-^ and MyD88^-/-^ C57BL/6 mice and prepared as described previously [16]. RAW264.7 macrophages were cultivated in DMEM (Dulbecco’s Modified Eagle medium) with 10% fetal bovine serum. The cells were seeded in 6-well culture dishes and then infected with *E*. *faecalis* E99 strain for 1 h or 5 h at an MOI of 10. For NF-κB and MAPKs inhibition experiments, RAW264.7 cells were pretreated for 0.5 h with one of the following inhibitors: MG132 (10 μM), SB239063 (10 μM), SP600125 (50 μM), PD98059 (25 μM), BAY 11–7082 (20 μM) or cytochalasin D (2.5 μg/ml) before infecting with E99 for 1 h or 5 h. These inhibitors were dissolved in DMSO and the control groups were treated with DMSO alone. To separate bacteria from macrophages, *E*. *faecalis* E99 cells were added to a monolayer of RAW264.7 cells on the top of a Transwell semipermeable filter (Corning Costar, Lowell, MA). To investigate the response of *E*. *faecalis* E99 infected RAW264.7 cells to LPS, RAW264.7 cells were infected with E99 for 1h, the cells were washed thrice with PBS and further incubated for 1 h, 3 h or 12 h with medium containing vancomycin and gentamicin to kill the extracellular bacteria before stimulation with LPS (1 μg/ml) for 3.5 h. The supernatant was collected to analyze TNF-α concentration by ELISA.

### Analysis of cytokine expression by reverse transcription (RT)-PCR and ELISA

For RT-PCR, the RNA was extracted from treated or untreated cells with an RNeasy Mini kit (QIAGEN, Valencia CA) and dissolved in DEPC-treated water. cDNA was generated with randomly primed RNA using iScript cDNA synthesis kit (Bio-Rad Laboratories, Hercules, CA) and used as template to measuring the levels of iNOS, TNF-α, IL-1β, INF-γ, arginase, IL-4 and actin. The supernatants collected from treated or untreated cells were used to detect the protein levels of TNF-α with an enzyme-linked immunosorbent assay (ELISA) kit (R&D Systems, Minneapolis, MN). Statistical significance was determined using Student’s *t* test.

### Western blot

RAW264.7 cells or BMDM were infected with *E*. *faecalis* E99 strain as described above, then washed with cold PBS and placed on ice. The cells in each well were harvested by scraping into lysis buffer consisting of 50 mM Tris (pH 7.4), 1.0% NP-40, 150 mM sodium chloride, 1 mM EDTA, 1 mM Sodium Fluoride, 1 mM Sodium Orthovanadate and 1 mM PMSF for 15 min. The protein concentration in the supernatant of the lysate was determined using a Bio-Rad protein assay after the lysate was centrifuged at 14,000 X g for 10 min. Equal amounts of protein (25–50 μg) was subjected to polyacrylamide gel electrophoresis on either 10 or 12% gels by using a Bio-Rad Mini PROTEAN II apparatus and transferred electrophoretically onto a PVDF membrane. After being blocked with 5% skim milk, the PVDF membranes were first incubated with MAPKs and NF-κB antibodies (dilution, 1:1,000) in 3% skim milk at 4°C overnight, followed by appropriate secondary antibodies conjugated to horseradish peroxidase (dilution, 1:3000). The primary antibodies used were Phospho-NF-κB p105, Phospho-NF-κB p65, IκBα, Phospho-p38 (Thr180/Tyr182), Phospho-ERK (Thr202/Tyr204), Phospho-JNK (Thr183/Tyr185) rabbit antibodies (Cell Signaling Technology, Beverly, MA), iNOS rabbit antibodies (Abcam, Cambridge, MA), IL-1β goat antibodies (R&D Systems, Minneapolis, MN, USA). Following three washes with TBST, the membranes were developed by using a chemiluminescent detection kit (Thermo Scientific, Rockford, IL).

### Bacterial phagocytosis assay

To determine the levels of phagocytosis of *E*. *faecalis* E99 by macrophages, E99 transformed with plasmid pMV158GFP to create GFP-expressing strain E99 (E99GFP) was used to infect RAW264.7 cells. The intracellular bacterial number was measured by flow cytometry or immunofluorescence staining or plate counting. RAW264.7 cells were seeded in 4 well chamber slides (BD Falcon) or in 24-well plates and cultured in DMEM with 10% fetal bovine serum. When the cells were almost confluent, they were cultured in serum-free DMEM and infected with E99GFP for 1 h. To investigate the effect of different inhibitors on the phagocytosis of *E*. *faecalis* by macrophages, RAW264.7 cells in DMEM were pretreated with cytochalasin D (2.5 μg/ml), PD98059 (25 μM), SB239063 (10 μM), or SP600125 (50 μM) for 0.5 h before infection with E99GFP for 1 h. The cells were washed thrice with PBS and then subjected to flow cytometry analysis using an Accuri C6 flow cytometer and immunofluorescence staining as described [17]. The anti-*E*. *faecalis* serum was produced by injection of heat-killed *E*. *faecalis* into SPF New Zealand white rabbits. Generation of rabbit antiserum to Esp has been previously described [18]. To investigate the influence of rabbit antiserum against whole *E*. *faecalis* or *E*. *faecalis* surface protein (Esp) on the phagocytosis of *E*. *faecalis* by macrophages, *E*. *faecalis* was preincubated with rabbit preimmune sera, serum against whole-cell enterococcal antigens or anti-Esp serum (diluted 1:50) for 30 min before infecting macrophages. For immunofluorescence staining, the treated cells were washed twice before incubating in DMEM containing 0.1% anti-*E*. *faecalis* rabbit antiserum for 15 min at 25°C, then the slides were washed twice with PBS and fixed with 4% paraformaldehyde for 5 min. The cells were rinsed twice with PBS before incubation with CF-594 labeled fluorescent anti-rabbit antibody for 30 min, to stain the extracellular bacteria. The cells were washed five times with PBS and then stained with DAPI. Cells were viewed and images acquired by using an Olympus IX73 fluorescence microscope. To quantify the viable bacteria in RAW264.7 cells at 1 h post infection in the above mentioned conditions, the cells were lysed by adding one-tenth of the volume of a saponin cell lysis solution to release intracellular bacteria according to the method described earlier [19]. The number of viable bacteria was enumerated by dilution and plating and expressed as CFU per 10^5^ macrophages.

### Statistical analysis

Unless otherwise noted, the differences between groups were analyzed using Student’s *t* test when only two groups were compared. The differences were considered statistically significant at p<0.05.

## Results

### Enterococcal infection induces M1 type proinflammatory cytokine expression

**Fig 1:**
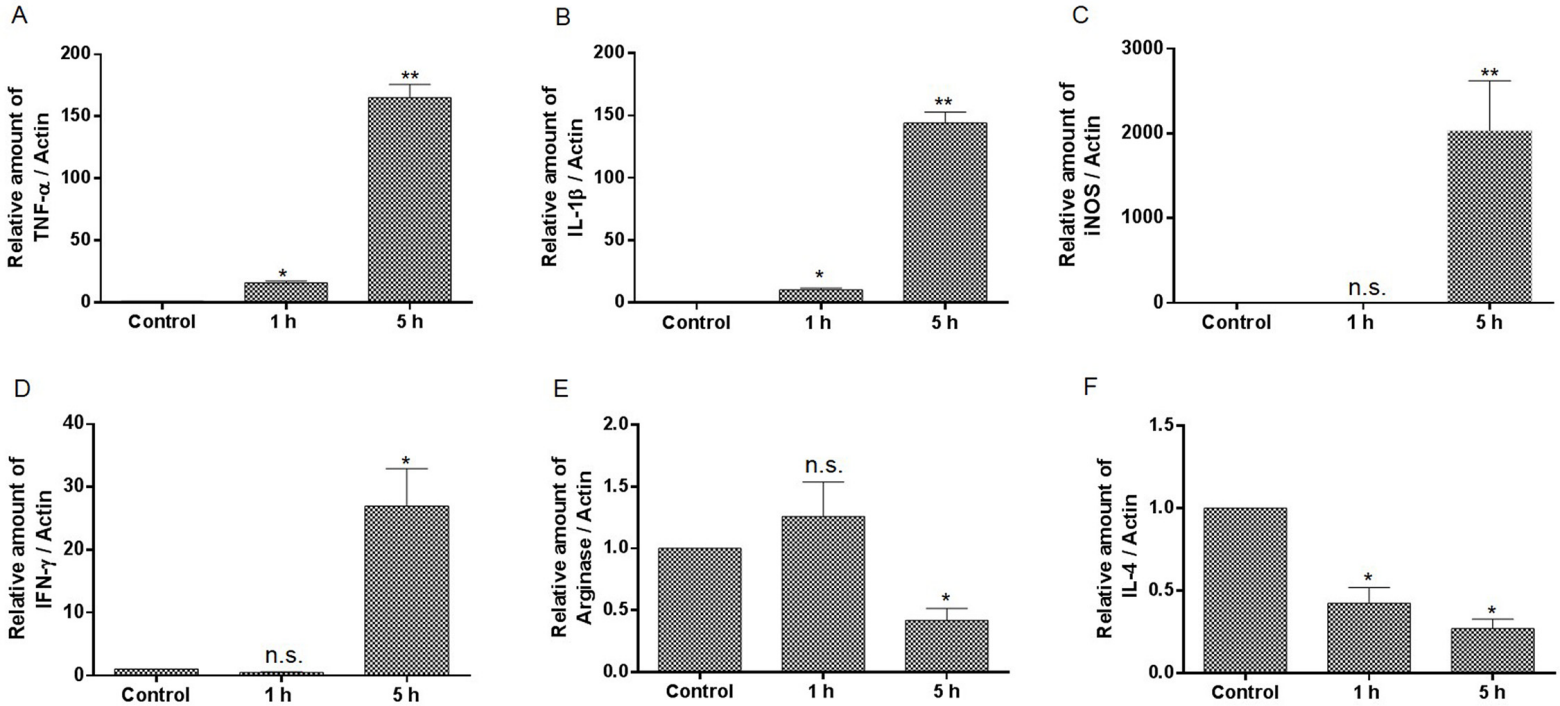
The expression of cytokines and other inducible mediators during BMDM infection by *E*. *faecalis*. BMDM were infected with *E*. *faecalis* E99 for 1 or 5 h before mRNA extraction and analysis by RT-PCR. The values are normalized to actin and expressed as the fold change relative to uninfected cells (Control). *, p<0.05; **, p<0.01 represent statistically significant difference compared to BMDM without infection; n.s., not statistically significant compared to BMDM without infection.

To investigate the effect of enterococcal infection on macrophage polarization, the expression of M1 and M2 cytokines were measured by RT-PCR. The mRNA of TNF-α, IL-1β, iNOS and INF-γ were measured as cytokines expressed by M1 type macrophages, whereas arginase and IL-4 mRNA were monitored as M2 phenotype-related cytokines. We treated bone marrow derived macrophage (BMDM) with *E*. *faecalis* E99 strain for 1 or 5 h, and found that the level of TNF-α, IL-1β, iNOS and INF-γ were significantly increased, while the M2-related cytokines including arginase and IL-4 gradually declined during enterococcal infection (Fig 1). The cytokines expression of TNF-α, iNOS and arginase were also measured in murine macrophage cell line RAW264.7 during *E*. *faecalis* infection, and showed similar increased expression of TNF-α and iNOS (S1 Fig).

### Enterococcus-induced proinflammatory cytokine expression is dependent on MAPKs and NF-κB activation

**Fig 2:**
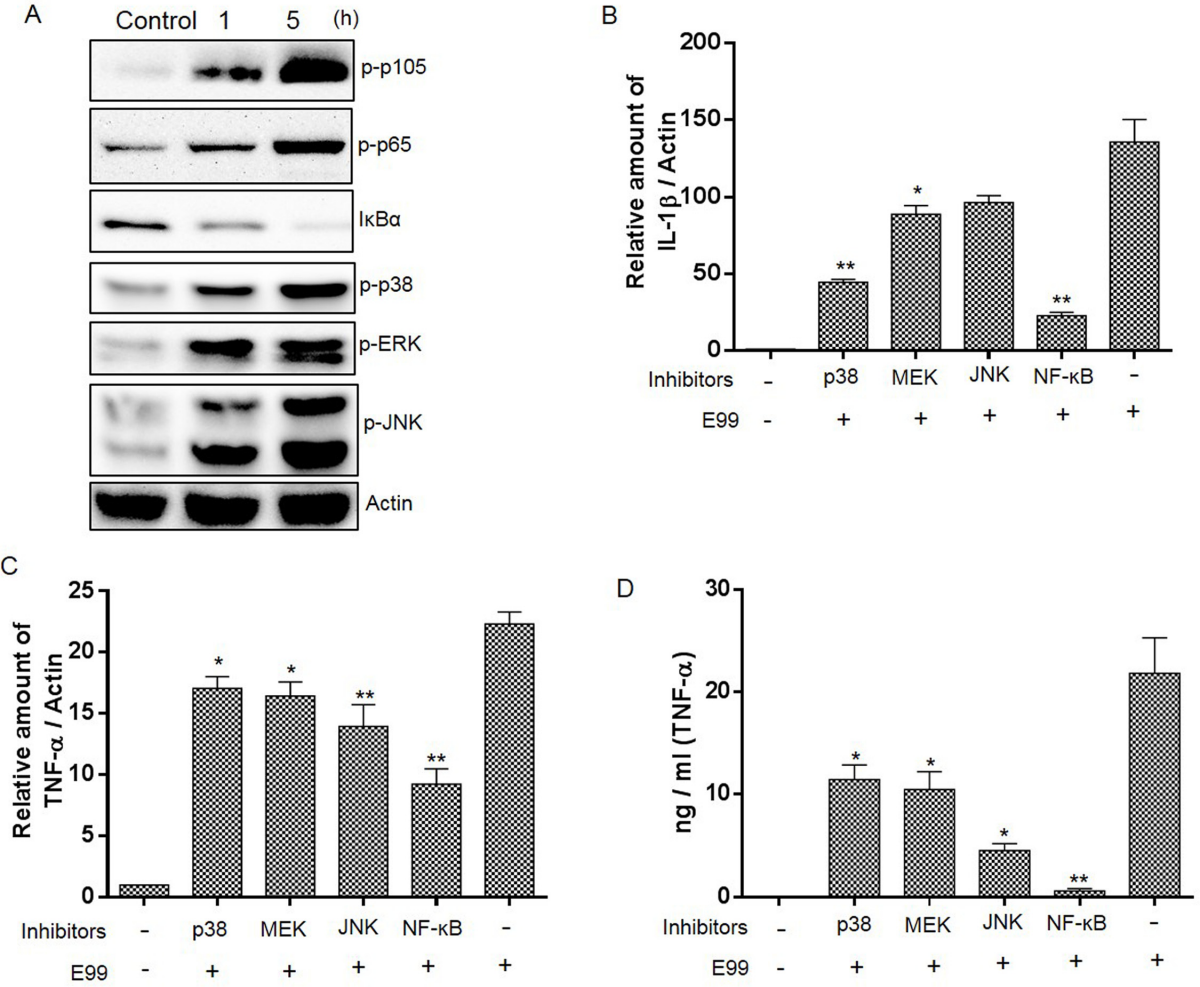
NF-κB and MAPKs are involved in *E*. *faecalis*-induced upregulation of cytokine expression. (A) RAW264.7 cells were infected with E99 at MOI of 10 for indicated times, then collected for Western blot analysis. (B&C) Cells were pretreated with the inhibitors of p38 (SB239063), JNK (SP600125), MEK (PD98059) or NF-κB (MG132) or with vehicle (DMSO) alone for 0.5 h before being infected with E99 for 5 h. The mRNA levels of TNF-α, IL-1β and actin in macrophages were measured by RT-PCR and the values were normalized to actin and expressed as the fold change relative to uninfected cells (Control). (D) TNF-α in the culture supernatants was measured by ELISA. *, p<0.05; **, p<0.01 represents statistically significant difference compared to RAW264.7 cells infected with E99 alone.

The NF-κB and MAP kinase (MAPK) signaling pathways play an important role in regulating the expression of cytokines including TNF-α, iNOS and IL-1β [20,21]. To investigate the contribution of NF-κB and MAPK to proinflammatory cytokines expression by macrophage during enterococcal infection, we first wanted to know the status of NF-κB and MAPK during enterococcal infection. The analysis of NF-κB activation was conducted by measuring the phosphorylation of p105 and p65 or degradation of IκB-α in RAW264.7 cells during infection. Our results showed that there was a significant increase in the phosphorylation of p105 and p65 in macrophages during enterococcal infection (1 h and 5 h) compared to pre-infection levels (Fig 2A). Consistent with this, the total IκB-α protein levels dropped during enterococcal infection (Fig 2A). The assessment of MAPKs activation was conducted by examining the phosphorylation of ERK, p38, and JNK by Western blot, and showed that enterococcal infection could lead to activation of all three major groups of MAPKs (Fig 2A). Similar to RAW264.7 cells, we also observed the activation of NF-κB and the three major groups of MAPKs during enterococcal infection in BMDM (S2 Fig). To elucidate the role of NF-κB and MAPKs in the expression of cytokines in macrophage in response to *E*. *faecalis* infection, we pretreated RAW264.7 cells with the inhibitors of NF-κB, MEK, P38 or JNK for 0.5 h, and then infected with E99 for 5 h. The expression of TNF-α and IL-1β at the transcriptional level was measured by quantitative RT-PCR, and the change in protein level of TNF-α was confirmed by ELISA. As shown in Fig 2B and 2C, inhibition of MEK, P38 and JNK could impair the expression of TNF-α and IL-1β at the mRNA level, with the only exception that inhibition of JNK could not significantly decrease the mRNA of IL-1β during infection. TNF-α and IL-1β expression were also significantly decreased due to inhibition of NF-κB by MG132 (Fig 2B and 2C) or by BAY 11–7082 (S2 Fig). The modulation of TNF-α secretion at the protein level by NF-κB and MAPKs was also confirmed by ELISA (Fig 2D). Together, these data implied that NF-κB plays a major role in controlling the expression of proinflammatory cytokines, and the three main MAPKs pathways were also involved in the production of cytokines during enterococcal infection.

### Contact of *E*. *faecalis* with macrophage is important to induce NF-κB and MAPKs activation and cytokine expression

**Fig 3:**
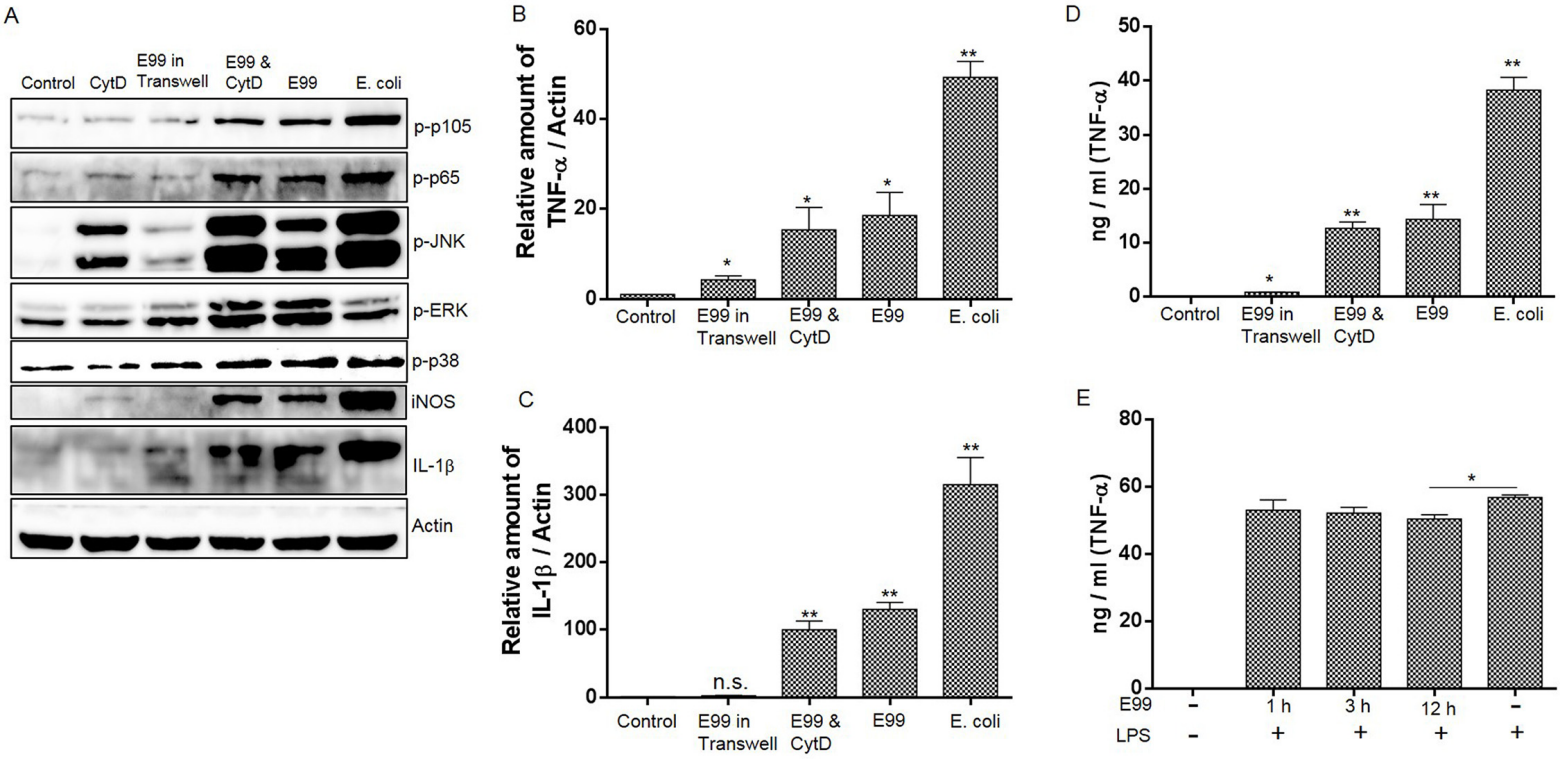
The effect of *E*. *faecalis* on NF-κB and MAPK activation and cytokine expression. This experiment included the following groups: (1) RAW264.7 cells alone; (2) RAW264.7 cells infected with *E*. *faecalis* E99; (3) RAW264.7 cells infected with *E*. *coli*; (4) *E*. *faecalis* E99 were added on the top of a Transwell semipermeable filter separating the bacteria from RAW264.7 cells; (5) the RAW264.7 cells were pretreated with CytD for 0.5 h before being infected with *E*. *faecalis* E99 for 5 h. The cells were collected to analyze the activation of NF-κB and MAPK by Western blot (A), or to measure the mRNA level of TNF-α (B) and IL-1β (C) by RT-PCR. (D) The supernatant from treated cells above were used to measure the concentration of TNF-α by ELISA. *, p<0.05; **, p<0.01 represent statistically significant difference compared to RAW264.7 cells without infection; n.s., not statistically significant compared to RAW264.7 cells without infection. (E) RAW264.7 cells were infected with E99 for 1h, cells were washed thrice with PBS and further incubated with medium containing vancomycin and gentamicin to kill the extracellular bacteria for 1 h, 3 h or 12 h before stimulation with LPS (1μg/ml) for 3.5 h. The supernatants were collected for analysis of TNF-α concentration by ELISA. *, p<0.05.

We next investigated the aspects of *E*. *faecalis* that may induce NF-κB and MAPK activation and cytokine expression. To test whether physical contact between bacteria and RAW264.7 cells was important for the proinflammatory cytokine expression, a semi-permeable cell culture insert was used to separate the live bacteria from macrophages. The results showed that separation of bacteria to prevent contact with RAW264.7 cells almost completely abolished NF-κB activation and could also significantly impair MAPK activation (Fig 3A). There was a slightly increased expression of TNF-α during this process, implying that effectors secreted by enterococci may induce TNF-α expression (Fig 3B and 3D). We then examined the role of bacterial internalization in NF-κB and MAPK activation and proinflammatory cytokine expression by inhibition of *E*. *faecalis* phagocytosis using cytochalasin D (CytD). Treatment of RAW264.7 cells with CytD alone could block the phagocytosis of enterococci by RAW264.7 cells and did not significantly affect NF-κB and MAPK activation with the exception that JNK was phosphorylated during CytD treatment (Fig 3A). Inhibition of phagocytosis by CytD did not affect the expression of TNF-α and IL-1β during enterococcal infection (Fig 3B, 3C and 3D). To further examine the role of internalized enterococci in the inflammatory response of macrophages, RAW264.7 cells were infected with *E*. *faecalis* for 1 h, then external *E*. *faecalis* were killed by gentamicin & vancomycin. When the RAW264.7 containing internalized *E*. *faecalis* were stimulated with LPS, it revealed an equivalent or slight decrease of TNF-α secretion, compared with LPS stimulated RAW264.7 cells not containing internalized *E*. *faecalis* (Fig 3E). These results implied that the activation of NF-κB and MAPK and subsequent cytokine expression is independent of enterococcal phagocytosis, and physical contact of enterococci is pivotal for activation of host cell signaling. Compared with *E*.*coli* infection, *E*. *faecalis* induced significantly lower levels of NF-κB and JNK activation in macrophages during infection, which is consistent with the lower level of TNF-α and IL-1β expression, and suggested that *E*. *faecalis* is only a modest inducer of inflammation during infection. Also it was observed that *E*. *faecalis* induced modest level of iNOS expression compared with *E*. *coli* infection (Fig 3A).

### MyD88 plays a central role in inducing NF-κB and MAPK activation and inflammation during *E*. *faecalis* infection

**Fig 4:**
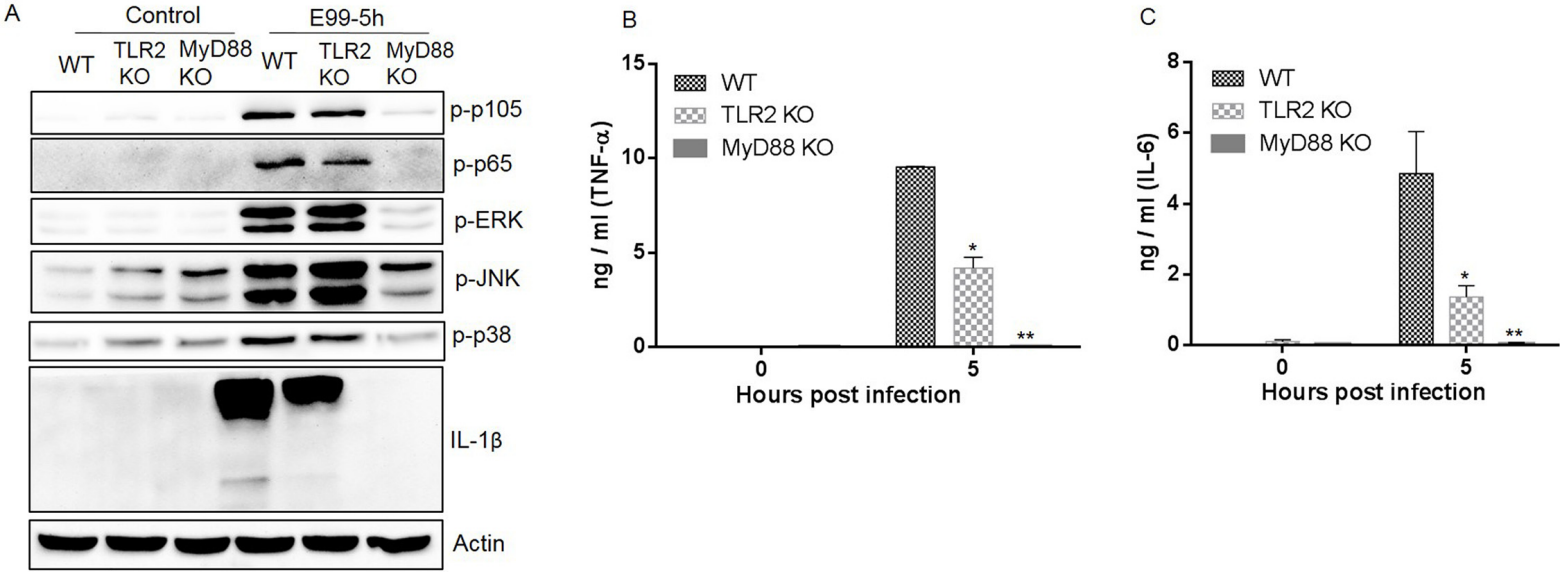
The roles of TLR2 and MyD88 in NF-κB and MAPK activation and cytokine secretion during *E*. *faecalis* infection. Wild type (WT), TLR2^-/-^ (TLR2 KO) and MyD88^-/-^ (MyD88 KO) BMDM were infected with E99 at a MOI of 10 for 5 h, the cells were then lysed and subjected to Western blot analysis (A). The supernatants were collected to measure the concentration of TNF-α (B) and IL-6 (C). *, p<0.05; **, p<0.01 represents statistically significant difference compared to WT BMDM infected with E99.

Although recent studies demonstrated the importance of TLR2 and MyD88 during enterococcal infection in mice [22], the regulation of NF-κB and MAPKs by TLR2 and MyD88 has not been explored. BMDM isolated from wild type, TLR2^-/-^ and MyD88^-/-^ C57BL/6 mice were infected with E99 for 5 h. Cell lysates analyzed by Western blot showed that enterococcal infection induced almost no phosphorylation of MAPKs and NF-κB activation in MyD88^-/-^ macrophages, with the exception that JNK phosphorylation was partially MyD88-independent (Fig 4A). There was a slight decrease in the phosphorylation of p105 and p65 in TLR2^-/-^ BMDM compared with that in wild type BMDM. Surprisingly, the pattern of MAPK phosphorylation induced by enterococcal infection in TLR2^-/-^ macrophages was similar to the pattern observed in wild type macrophages (Fig 4A). TNF-α, IL-6 and IL-1β secretion was impaired in macrophages from TLR2^-/-^ mice, and abolished in MyD88^-/-^ macrophages, compared with macrophages from wild type mice (Fig 4A, 4B and 4C). These results suggested that activation of NF-κB and MAPKs, and proinflammatory cytokine expression in *E*. *faecalis* infected macrophages is partially dependent on TLR2 and is largely mediated via MyD88.

### The phagocytosis of enterococcus by macrophages is enhanced by preopsonization

**Fig 5:**
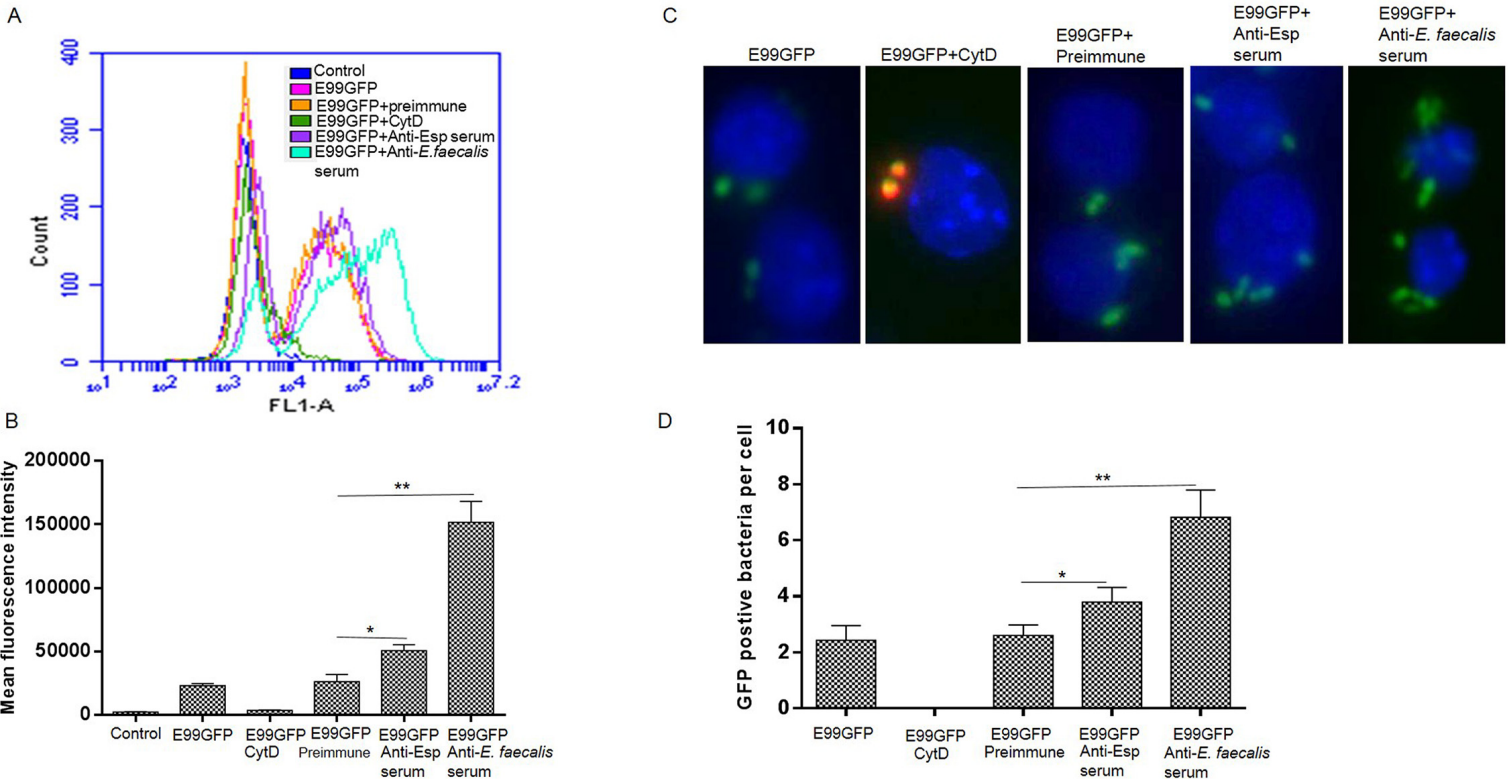
Phagocytosis of E99GFP by RAW264.7 cells is increased when *E*. *faecalis* is opsonized with serum against whole *E*. *faecalis* cells or Esp. (A) FACS analysis of phagocytosis of E99GFP under different conditions including: RAW264.7 cell without infection (Control), RAW264.7 cells infected with E99GFP (E99GFP); RAW264.7 cells pretreated with CytD before infecting with E99GFP (E99GFP+CytD); RAW264.7 cells infected with E99GFP pretreated with rabbit preimmune sera (E99GFP+preimmune), serum against Esp (E99GFP+Anti-Esp serum) or serum against whole-cell enterococcal antigens (E99GFP+ Anti-*E*. *faecalis* serum). (B) The mean fluorescence intensity of macrophages was measured by flow cytometry from A. (C) Representative immunofluorescence images showing phagocytosis of E99GFP by RAW264.7 cells under different treatments, Blue: DAPI, Green: E99GFP and Red: antibody against extracellular E99. (D) For each treatment, the number of E99GFP bacteria in at least twenty macrophages were counted and the mean number of intracellular E99GFP per cell was calculated *, p<0.05; **, p<0.01.

To investigate the phagocytosis of E99 by macrophages during infection, we examined the phagocytic index by infecting macrophages with GFP-expressing bacteria (E99GFP) at MOI of 10, 50, and 100 and allowing the infection to proceed for 30 min, 1 h and 2 h. Macrophages were then washed and examined by FACS. The results showed that there was an average of 10–60% of macrophages containing internalized *E*. *faecalis* under these infection conditions. The percentage of infected macrophages increased to 50% or more only at MOI of 50 for 2 h or 100 for 1 or 2 h (S3 Fig). Accordingly, an MOI of 100 and 1 h infection time was used to evaluate *E*. *faecalis* phagocytosis by RAW264.7 cells with FACS. Treatment of RAW264.7 cells with CytD, an actin-polymerization inhibitor, showed a complete inhibition of phagocytosis of E99GFP measured by FACS analysis (Fig 5A). Next, we investigated the effect of preopsonization of enterococci with serum raised against whole *E*. *faecalis* cells, and found that the uptake of *E*. *faecalis* by the macrophages increased significantly compared to those treated with preimmune serum (Fig 5A and 5B). The enterococcal surface protein (Esp) is expressed on the surface of most *E*. *faecalis* clinical strains including E99. To further investigate the contribution of Esp specific antibody for macrophage phagocytosis of enterococci, *E*. *faecalis* E99GFP was opsonized with polyclonal antibodies to Esp. The result showed that although phagocytosis was enhanced with bacteria opsonized with anti-Esp serum compared with bacteria opsonized with preimmune serum, it was significantly less than cells opsonized with antiserum to whole bacteria (Fig 5A and 5B). The use of immunofluorescence staining allowed discrimination between extracellular and internalized bacteria when we stained with an antibody against whole *E*. *faecalis*. Consistent with data from FACS, a blockade of phagocytosis of E99GFP was also observed in CytD-treated RAW264.7 cells and there was also an increase in phagocytosis of E99GFP opsonized with *E*. *faecalis*-positive serum or anti-Esp serum compared with preimmune serum (Fig 5C and 5D). The enhanced phagocytosis of E99 by macrophages, when *E*. *faecalis* is opsonized with serum against whole *E*. *faecalis* cells or Esp, was further confirmed by counting viable bacteria in macrophages at 1 h post E99 internalization (S4 Fig), suggesting the phagocytosis of *E*. *faecalis* correlated with Fc receptor-mediated phagocytosis.

### MyD88 and MAPK pathways play an important role in regulation of phagocytosis of enterococci by macrophages

**Fig 6:**
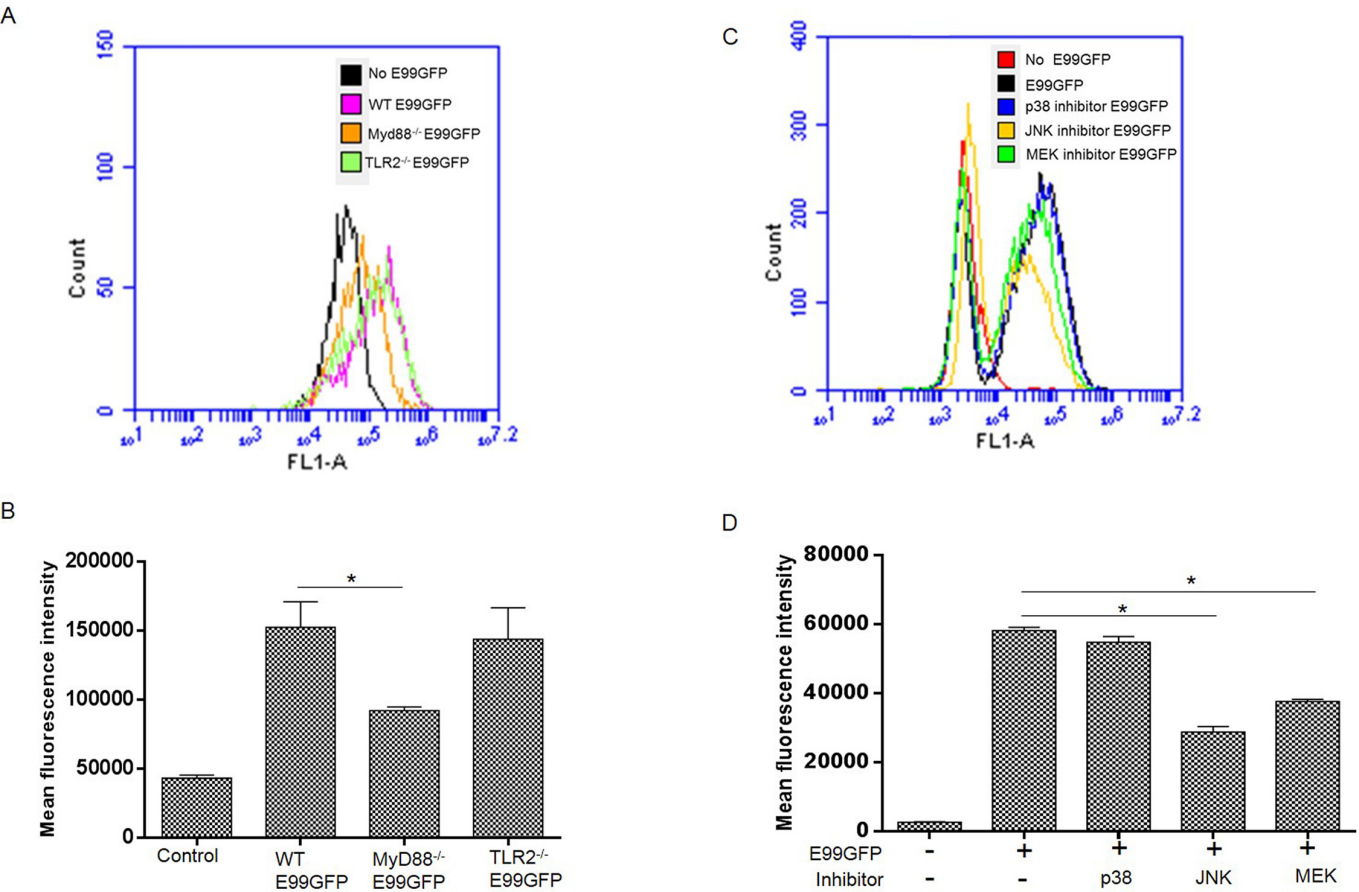
Impaired phagocytosis in the absence of MyD88, ERK and JNK signal pathway. (A) WT, TLR2^-/-^, MyD88^-/-^ BMDM were infected with E99GFP at a MOI of 100 for 1 h, then the cells were analyzed by FACS after washing thrice with PBS. Representative FACS histogram shows the phagocytosis of E99GFP by WT, TLR2^-/-^ and MyD88^-/-^ BMDM. (B) Mean fluorescence intensity of GFP from A. *, p<0.05. (C) RAW264.7 cells were pretreated with inhibitors of p38, JNK or MEK and then infected with E99GFP at MOI of 100 for 1 h. The cells were washed with PBS for three times before analysis by FACS. FACS histogram shows phagocytosis of E99GFP by RAW264.7 cells with different treatments. (D) Mean fluorescence intensity at 1 hour after phagocytosis of E99GFP by RAW264.7 cells from C. *, p<0.05;

To reveal the role of TLR2 and MyD88 in phagocytosis of enterococci, we compared the phagocytosis of *E*. *faecalis* by macrophages from wild type, TLR2^-/-^ and MyD88^-/-^ mice. Since an MOI of 100 and 1 h infection time resulted in a reasonable percentage of infected macrophages for analysis by FACS, wild type, TLR2^-/-^ and MyD88^-/-^ macrophages were infected with GFP expressing E99 (E99GFP) under these conditions. It was observed that MyD88^-/-^ macrophages showed significant impairment of internalization of *E*. *faecalis*, while phagocytosis of enterococci by TLR2^-/-^ macrophages was not significantly impaired (Fig 6A and 6B). To determine the effect of inhibition of MAPKs on phagocytosis of enterococcus, RAW264.7 cells were pretreated with p38 MAPK, JNK and MEK inhibitors before being infected with E99GFP. Specific inhibition of MEK, JNK, but not p38 MAPK decreased the ability of macrophages to efficiently phagocytize *E*. *faecalis*, as compared with control (Fig 6C and 6D). The effect of JNK and MEK signaling on phagocytosis of *E*. *faecalis* by macrophages was also confirmed by enumerating the viable intracellular bacteria in macrophages by plate counting (S4 Fig). Based on our studies which showed that there was a significant impairment of MAPKs activation in MyD88^-/-^ BMDM during enterococcal infection, we speculated that the impaired phagocytosis of enterococci by MyD88^-/-^ BMDM may be at least partially due to the reduction of ERK and JNK activation during enterococcal infection.

## Discussion

Macrophages are considered to be major host effectors in the defense against bacterial infection. Confirmation of their ability to respond to Gram-positive and Gram-negative bacteria has been achieved through numerous studies with lymphocyte-free bone marrow-derived mononuclear phagocytes and with murine macrophage cells such as RAW264.7 and J774.1 [23,24]. These studies have shown that while macrophages do recognize these organisms in the absence of components of the host specific immune system, the pattern of their response could vary considerably depending on the type of bacteria.

Although enterococci are generally considered harmless commensals in the normal host, the dramatic increase in hospital-acquired infections, especially in immunocompromised hosts, has been brought about by a combination of high level antibiotic resistance within this genus coupled with the acquisition of virulence traits that allows them to evade host defenses. Recently it was reported that *E*. *faecalis* infection can lead to severe sepsis or septic shock in immunocompromised patients, burn patients and thermally injured mice by modulation of the host systemic inflammatory response [25,26]. Employing a mouse model of *E*. *faecium* peritonitis, a previous study demonstrated that depletion of resident peritoneal macrophages caused a clear delay in peritoneal clearance and was associated with increased systemic dissemination of bacteria [6]. In our studies, we extended these earlier observations to investigate the interaction between macrophages and a clinical *E*. *faecalis* strain *in vitro*, and show that while *E*. *faecalis* infection could induce a M1 type inflammatory cytokines gene expression, it was modest compared to that induced by a non-pathogenic *E*. *coli* strain. While polarization of macrophages to M1-type during bacterial infection is associated with microbicidal and inflammatory activities [27–29], excessive or prolonged M1 polarization can lead to tissue injury and contribute to pathogenesis. The modest M1 type inflammatory cytokine expression during *enterococcal* infection may partially explain why low doses of enterococci could be quickly eliminated from the infected mice with no serious pathology in the tissue in the peritonitis infection model [22,30].

By using MyD88 knockout and TLR2 knockout mice, it was previously shown that MyD88 contributes to the effective clearance of *E*. *faecium* during peritonitis at least in part via TLR2/MyD88 signaling pathway and by facilitating neutrophil recruitment to the site of infection *in vivo* [22]. However, it was observed that enterococcal infection induced a transient and low level cytokine expression response in the peritonitis infection model, which made it difficult to decipher the mechanism of cytokine regulation *in vivo* [31]. By using an *in vitro* approach to reveal the key effectors and related host signaling pathway that participate in the induction of proinflammatory response during *E*. *faecalis* infection, our study here found that cytokine expression is regulated by NF-κB and MAPK pathways, with MyD88 as the key adaptor to regulate the proinflammatory response. Although the previous study showed that MyD88 or TLR2 did not seem to contribute to phagocytosis of *E*. *faecium* by peripheral blood neutrophils [22], our present study revealed that MyD88^-/-^ bone marrow derived macrophage (BMDM) showed significant impairment of internalization of *E*. *faecalis* compared with wild type BMDM. These differences in observations between the two studies could be due to differences in the host cell types and/or the different bacterial species and effector molecules involved.

A recent study showed that *E*. *faecalis* isolated from healthy infants can suppress inflammatory responses in intestinal epithelial cells [32]. While this study demonstrated that the involvement of MAPK signaling through P38, P-JNK and C-JUN appeared to be central to the effect of *E*. *faecalis* on these cells as well, the observed results suggested that *E*. *faecalis* suppressed activation of P38, P-JNK and C-JUN with no effect on ERK phosphorylation [32]. In our present study, we showed that macrophages were activated to induce proinflammatory cytokines expression during enterococcal infection. The different response of macrophage and epithelial cells to *E*. *faecalis* infection may reflect the cell type specific responses during stimulation. The different *in vitro* response to *E*. *faecalis* infection between epithelial cells and macrophages may also have implications in the control of *E*. *faecalis* infection. During enterococcal colonization in the intestine, induced anti-inflammatory responses in epithelial cells may help to maintain intestinal hemostasis, while under situations such as translocation across the epithelial barrier and encounter with immune cells such as macrophages, induced proinflammatory responses may aid the host in clearing the infection from extraintestinal sites.

The response of phagocytes including macrophages, dendritic cells and neutrophils can be multifaceted and have numerous roles in immunity and inflammation. Effective clearance of pathogenic microorganisms by phagocytes involves the initial detection through surface receptors followed by uptake and killing. Most phagocytes produce or direct the production of proinflammatory cytokines and chemokines that orchestrate local and systemic inflammatory responses. The activation of the inflammatory response by phagocytes could rely on the cell surface receptors such as TLR2 and TLR4 which could recognize bacteria cell surface components LTA and LPS respectively, or intracellular receptors such as TLR9 could recognize unmethylated bacterial CpG DNA to induce proinflammatory cytokine expression. Our results showing that the contact of *E*. *faecalis* with macrophages rather than internalized bacteria plays an important role in NF-κB and MAPKs activation and the subsequent cytokine expression, suggests that the pathogen associated molecular patterns recognized by phagocytes during enterococcal infection are largely attributable to the cell surface components of enterococci.

The enterococcal cell surface is rather complex consisting of peptidoglycan (PG), cell wall associated and anchored proteins, lipoteichoic acid (LTA), wall-associated teichoic acid (WTA), enterococcal polysaccharide antigen (Epa) and capsular polysaccharide (Cps). While the precise contributions of each of these structurally different molecular entities at the bacterial cell surface to the recognition by phagocytes and shaping of the host immune response remains to be established, it is known that LTA is an immunodominant molecule on the cell surface. In general LTAs are comprised of glycerol phosphate polymers of repeating units esterified with D-alanine, α-D-N-acetylglucosamine and no substituent [33]. The number of repeating units and the content of D-alanine seems to correlate with the inflammatory potential of LTA. Lipoteichoic acid from *E*. *faecalis* has been shown to have a unique structure [34] and contribute partially to *E*. *faecalis*-induced inflammatory responses through stimulation via TLR2 [35,36]. Consistent with these observations, when we examined the response of TLR2^-/-^ BMDM to *E*. *faecalis* infection, we found that TLR2 deficiency could only partially decrease the inflammatory response in macrophage, implying that activation of macrophage during enterococcal infection could also be through TLR2-independent signaling pathways. In contrast, deficiency of host cell adaptor protein MyD88 could almost completely abolish the activation of NF-κB and MAPK pathways, and subsequent cytokines expression by macrophages during enterococcal infection.

Phagocytosis by macrophages relies on the complex interplay between the cell and the microorganism in a tightly regulated process. It can either be direct via the pathogen recognition receptors (PRRs) on the host cell surface such as the mannose receptor (MR) recognizing surface carbohydrates, peptidoglycans on the pathogen or, indirect via opsonization of the microbe by host factors such as IgG or components of the complement system prior to phagocytosis [37]. In our study, we found that enterococcus internalization is dependent on actin dynamics, and the internalization process could be regulated by MyD88-dependent signaling pathway. Opsonization of *E*. *faecalis* with the serum generated against whole bacteria or surface protein Esp serum could significantly increase the phagocytosis of *E*. *faecalis* by macrophage indicating that FcγR-mediated phagocytosis plays an important role during enterococcus internalization by macrophages. In summary, the findings from this study adds to our growing knowledge regarding enterococcal-host relationships, particularly with key immune cells, and has important implications for understanding the dichotomous roles of enterococci both as a commensal and as an opportunistic pathogen.

## Supporting Information

S1 Fig The mRNA levels of iNOS, TNF-α and arginase in *E*. *faecalis* E99-infected RAW264.7 cells at 1 or 5 h postinfection analyzed by RT-PCR. The values are normalized to actin and expressed as the fold change relative to uninfected cells (Control).*, p<0.05 represent statistically significant difference compared to RAW264.7 cells without infection; n.s., not statistically significant compared to RAW264.7 cells without infection.
(TIF)

S2 Fig The activation of NF-κB and MAPKs in BMDM and regulation of cytokines expression by NF-κB during *E*. *faecalis* infection. (A) BMDM cells were infected with E99 at a MOI of 10 for indicated times and then the cells were collected to analyze the activation of NF-κB and MAPKs by Western blot. (B&C) RAW264.7 cells were treated with NF-κB inhibitor (BAY 11–7082) for 30 min before infected with E99 at a MOI of 10 for 5 h. The mRNA levels of TNF-α (B) and IL-1β (C) in *E*. *faecalis* E99-infected RAW264.7 cells or uninfected cells were analyzed by RT-PCR. *, p<0.05; **, p<0.01.
(TIF)

S3 Fig The internalization of *E*. *faecalis* E99 by RAW264.7 macrophages. RAW264.7 cells were infected with E99GFP under different conditions and then the cells were washed thrice with PBS before analysis by FACS to calculate the percentage of RAW264.7 cells containing internalized *E*. *faecalis* E99.
(TIF)

S4 Fig Analysis of viable intracellular bacteria in macrophages by serial dilution and plating. RAW264.7 cells infected with E99 pretreated with rabbit preimmune sera (E99+preimmune), serum against Esp (E99+Anti-Esp serum) or serum against whole-cell enterococcal antigens (E99+ Anti-*E*. *faecalis* serum) at MOI of 10 for 1h (A), or RAW264.7 cells were pretreated with inhibitors of p38, MEK or JNK for 30 min and then infected with E99 at MOI of 10 for 1 h (B). The cells were washed with PBS for three times and the intracellular bacteria were quantified by serial dilution and plating. The number of viable bacteria was expressed as CFU per 10^5^ macrophages. *, p<0.05; **, p<0.01.
(TIF)
