# Supplementary figures and images for: Roles of TLR/MyD88/MAPK/NF-κB Signaling Pathways in the Regulation of Phagocytosis and Proinflammatory Cytokine Expression in Response to E. faecalis Infection
Source: PLoS One. 2015 Aug 28;10(8):e0136947. doi: 10.1371/journal.pone.0136947 (PMC4552673; doi:10.1371/journal.pone.0136947)

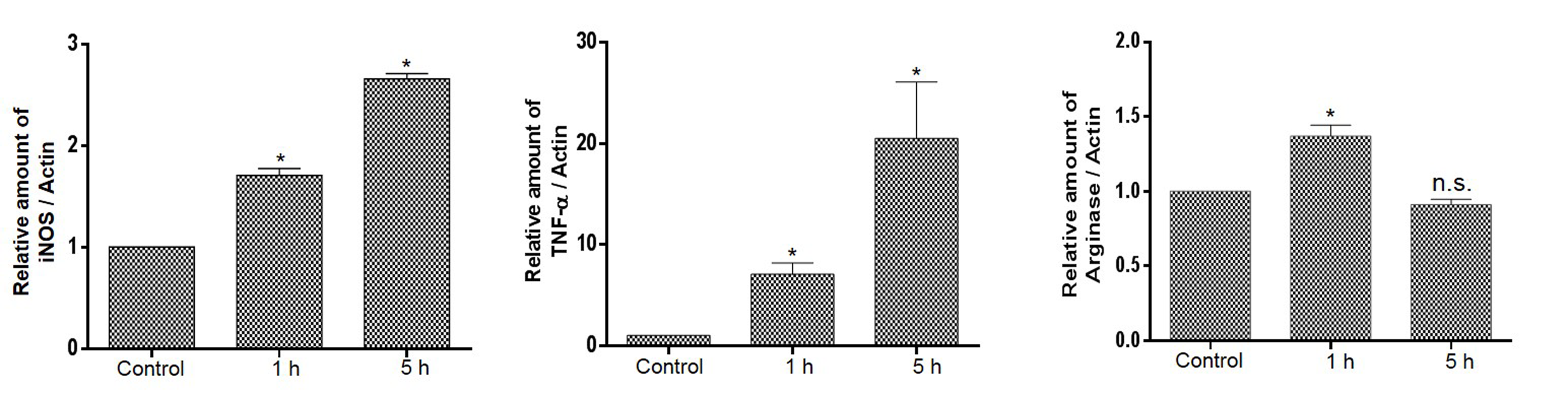

Supplement: S1 Fig — The values are normalized to actin and expressed as the fold change relative to uninfected cells (Control).*, p<0.05 represent statistically significant difference compared to RAW264.7 cells without infection; n.s., not statistically significant compared to RAW264.7 cells without infection. (TIF) [file pone.0136947.s001.tif]

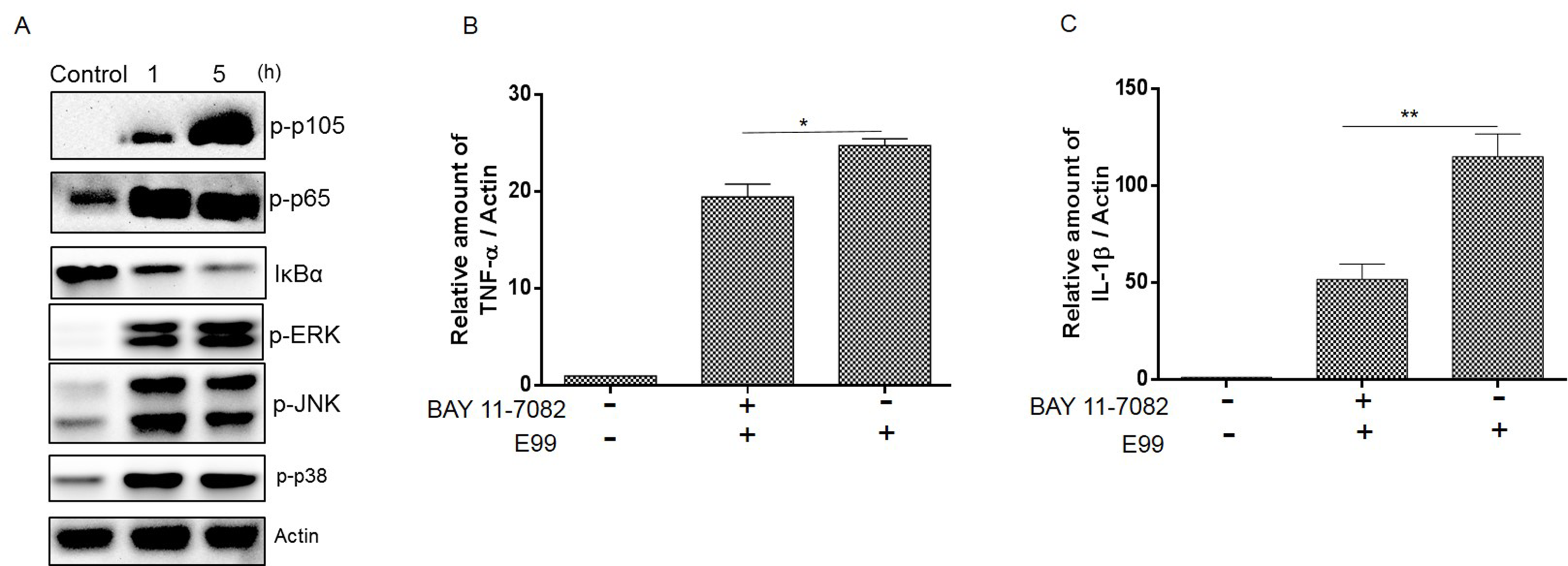

Supplement: S2 Fig — (A) BMDM cells were infected with E99 at a MOI of 10 for indicated times and then the cells were collected to analyze the activation of NF-κB and MAPKs by Western blot. (B&C) RAW264.7 cells were treated with NF-κB inhibitor (BAY 11–7082) for 30 min before infected with E99 at a MOI of 10 for 5 h. The mRNA levels of TNF-α (B) and IL-1β (C) in E. faecalis E99-infected RAW264.7 cells or uninfected cells were analyzed by RT-PCR. *, p<0.05; **, p<0.01. (TIF) [file pone.0136947.s002.tif]

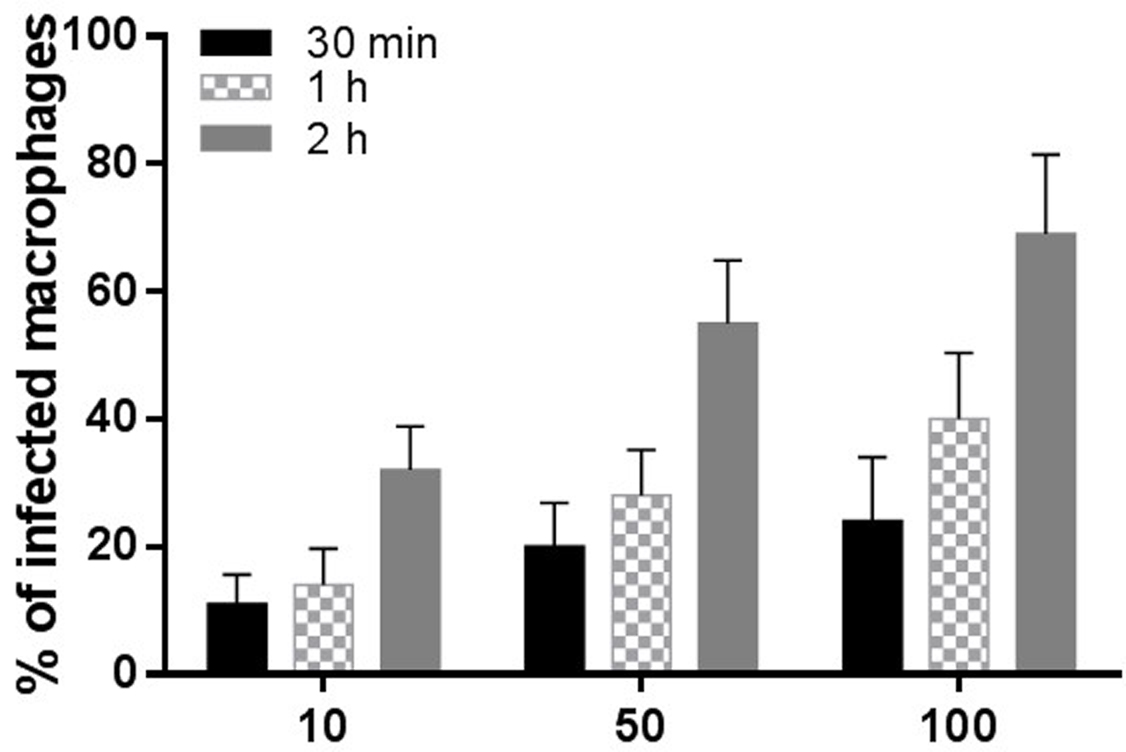

Supplement: S3 Fig — RAW264.7 cells were infected with E99GFP under different conditions and then the cells were washed thrice with PBS before analysis by FACS to calculate the percentage of RAW264.7 cells containing internalized E. faecalis E99. (TIF) [file pone.0136947.s003.tif]

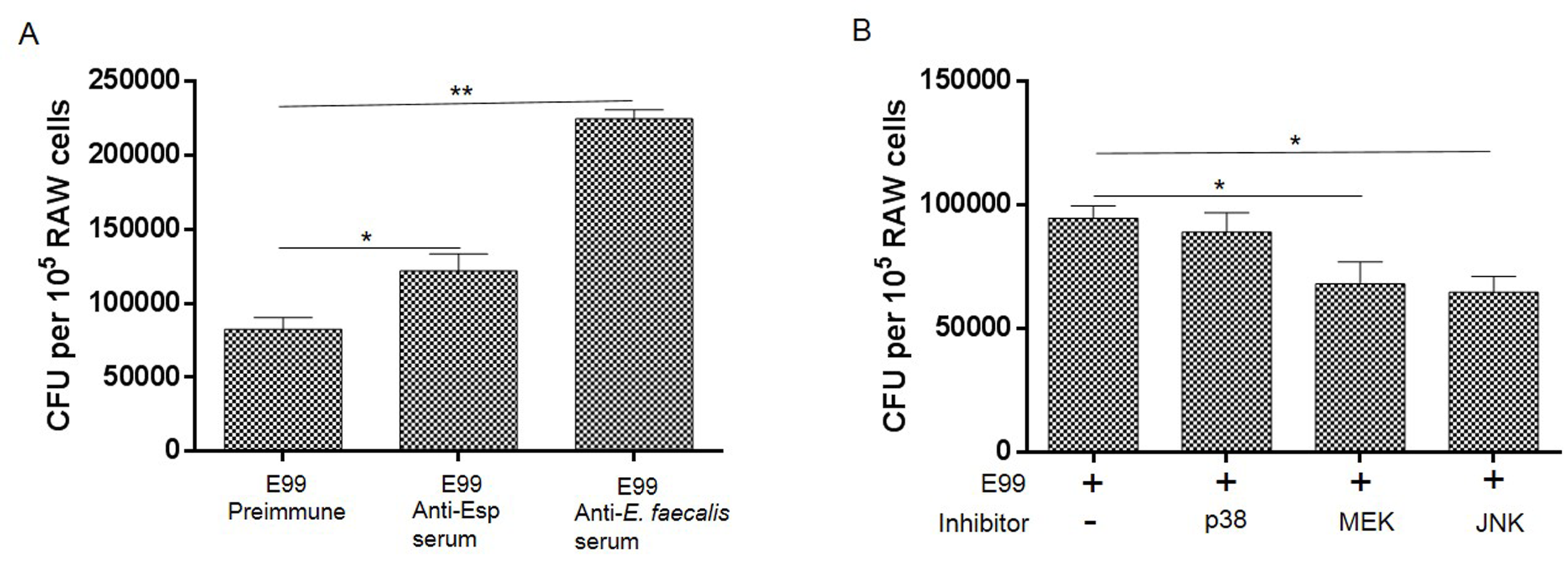

Supplement: S4 Fig — RAW264.7 cells infected with E99 pretreated with rabbit preimmune sera (E99+preimmune), serum against Esp (E99+Anti-Esp serum) or serum against whole-cell enterococcal antigens (E99+ Anti-E. faecalis serum) at MOI of 10 for 1h (A), or RAW264.7 cells were pretreated with inhibitors of p38, MEK or JNK for 30 min and then infected with E99 at MOI of 10 for 1 h (B). The cells were washed with PBS for three times and the intracellular bacteria were quantified by serial dilution and plating. The number of viable bacteria was expressed as CFU per 105 macrophages. *, p<0.05; **, p<0.01. (TIF) [file pone.0136947.s004.tif]
